# Aloe emodin disrupts *Candida albicans* mitochondrial iron homeostasis against its hyphal development and oral candidiasis

**DOI:** 10.1007/s00253-026-13740-1

**Published:** 2026-02-20

**Authors:** Jiawei Shen, Chuanli Zhang, Yifan Lin, Chunfei Zhang, Jingzhi Zhou, Yujie Zhou, Lichen Gou, Ga Liao, Zhuoli Zhu, Lei Cheng, Binyou Liao, Biao Ren

**Affiliations:** 1 State Key Laboratory of Oral Diseases, National Center for Stomatology, National Clinical Research Center for Oral Diseases, West China School of Stomatology, Sichuan University, Chengdu, 610041 Sichuan China; 2 Department of Hematology, West China Hospital, Sichuan University, Chengdu, 610041 Sichuan China; 3 School of Nursing, Beijing University of Chinese Medicine, Beijing, 100029 China; 4 Hospital of Stomatology, Guangdong Provincial Key Laboratory of Stomatology, Guanghua School of Stomatology, Sun Yat-Sen University, Guangzhou, 510055 Guangdong China; 5 Department of Geriatric Dentistry, West China Hospital of Stomatology, Sichuan University, Chengdu, 610041 Sichuan China; 6 Department of Operative Dentistry and Endodontics, West China School of Stomatology, Sichuan University, Chengdu, 610041 Sichuan China; 7 Department of Periodontics, West China School of Stomatology, Sichuan University, Chengdu, 610041 Sichuan China; 8 Tianfu Jiangxi Laboratory, Chengdu, 641419 Sichuan China

**Keywords:** Aloe emodin, *Candida albicans*, Antifungal activity, Mitochondrial damage, Oropharyngeal candidiasis

## Abstract

**Abstract:**

*Candida albicans*, a critical pathogen listed on the WHO fungal priority pathogens list, is a major cause of candidiasis and candidemia, particularly in immunocompromised populations. The lack of novel antifungal drugs and the increasing prevalence of drug resistance underscore the urgent need for new therapeutic strategies. By screening compounds derived from *Aloe vera* using hyphal induction assays, we identified aloe emodin (AE) as a potent inhibitor of *C. albicans* hyphal development. We demonstrate for the first time that AE, at concentrations of 50–100 μg/mL that do not affect fungal growth, significantly inhibits *C. albicans* hyphal development and its infections to oral epithelial cells without cytotoxicity. The confocal observation and following transcriptome and metabolome analysis demonstrate that AE localizes to fungal mitochondria and chelates iron, thereby disrupting iron homeostasis. These effects lead to mitochondrial dysfunction and metabolic reprogramming, particularly by impairing succinate dehydrogenase 2 activity, thereby reducing ATP and cAMP production. The decrease in cAMP levels leads to downregulation of the cAMP–PKA signaling pathway, a central regulator of hyphal development, ultimately suppressing hyphal growth. Mitochondrial function and metabolic assays, together with validation using mutants of the cAMP–PKA pathway, further confirmed the mechanism underlying the anti-hyphal activity of AE. AE at 50–100 μg/mL administered via drinking water also significantly reduced fungal burden and tissue damage in an oropharyngeal candidiasis model, which is similar with clinical antifungal nystatin. Collectively, our findings indicate that AE is a promising candidate for development as a therapeutic agent for oral candidiasis.

**Key points:**

• *Aloe emodin disrupts mitochondrial iron homeostasis to remodel metabolism in C. albicans.*

• *Aloe emodin downregulates the cAMP-PKA pathway to inhibit C. albicans hyphae.*

• *Aloe emodin functions as a novel candidate for treating infections caused by C. albicans.*

**Graphical Abstract:**

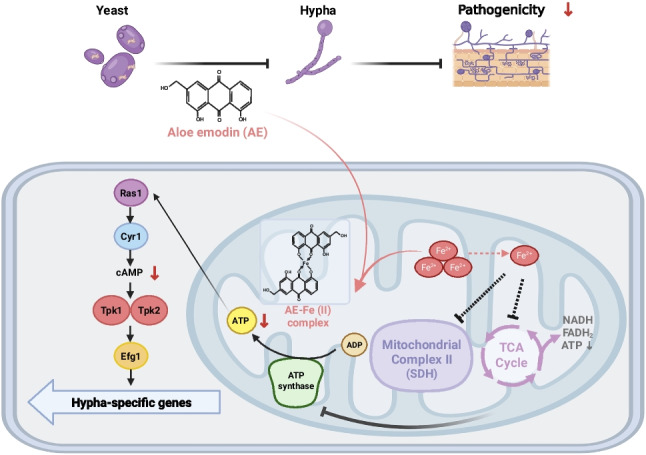

**Supplementary Information:**

The online version contains supplementary material available at 10.1007/s00253-026-13740-1.

## Introduction

*Candida albicans* is a predominant opportunistic pathogen within the human mycobiome (Huang et al. 2021; Khan et al. 2023). A steady increase in both the incidence and mortality of *C. albicans* infections has been observed (Giannella et al. 2025), and it has been listed as a “critical priority” pathogenic fungi by the World Health Organization (2022) (Parums 2022). In the oral cavity, *C. albicans* is the primary causative agent of oral candidiasis, the most prevalent oral fungal infection (Vila et al. 2020), and is also closely associated with the development of dental caries, periapical diseases, and periodontal diseases (Chen et al. 2020b; Yang et al. 2023). Currently, the main classes of clinical antifungal agents include azoles, polyenes, echinocandins, and flucytosine. Over the past 30 years, only one novel antifungal agent, ibrexafungerp, has been approved for the treatment of vulvovaginal candidiasis (Sobel et al. 2022). The lack of novel antifungal drugs, combined with the widespread and overuse of existing drugs, has contributed to the rapid emergence of antifungal drug resistance (Castanheira et al. 2016; Rhodes and Fisher 2019). Consequently, the development of novel, safe, and highly effective antifungal drugs has become an urgent priority.

In contrast to traditional antifungal agents that exert fungicidal or fungistatic effects, targeting fungal virulence represents a novel and practical therapeutic strategy (Vila et al. 2016). This approach significantly reduces the likelihood of drug-resistant mutations (Martínez et al. 2019), while preserving the balance of the native microbiome of the host (Cegelski et al. 2008). The major pathogenic virulence of *C. albicans* is highly dependent on the transition from yeast to hyphal growth (Sturtevant et al. 2013). This morphological switch is regulated by multiple signaling pathways, including the cyclic adenosine monophosphate–protein kinase A (cAMP–PKA) pathway, which governs fungal metabolic adaptation and responses to environmental stress. The cAMP–PKA signaling cascade plays a central role in sensing and responding to environmental cues that trigger hyphal growth in *C. albicans* (Chen et al. 2020a; Sudbery 2011). The mechanical force generated by *C. albicans* hyphae enables the fungus to penetrate epithelial barriers, facilitating tissue invasion and hematogenous dissemination (Arkowitz and Bassilana 2019; Liu et al. 2024). Adhesins that are specifically expressed during hyphal growth, including Als3 and Hwp1, play critical roles in enhancing fungal adherence to host cells (Schena et al. 2025). In addition, secreted aspartic proteases and the cytolytic peptide candidalysin promote host cell damage and immune evasion (Kumar et al. 2022; Nikou et al. 2022). Hyphal morphology is also essential for biofilm formation, conferring high levels of antifungal drug tolerance and contributing to persistent clinical infections (Sushmitha et al. 2023). Therefore, targeting *C. albicans* hyphal development represents a pivotal strategy for the discovery of new antifungal therapeutics.

Traditional Chinese medicine represents a rich resource for the development of antifungal therapies, particularly antivirulence agents. For example, artemisinin and its derivatives have demonstrated significant antifungal activity (Galal et al. 2005; Liang et al. 2023; Zhu et al. 2021). Artemisinin increases ergosterol content in the fungal cell membrane, thereby enhancing the binding affinity of polyene drugs and promoting fungal killing (Zhu et al. 2021). It can also disrupt *C. albicans* metabolism, leading to downregulation of the cAMP–PKA signaling pathway and inhibition of the yeast-to-hypha transition (Liang et al. 2023). *Aloe vera* has a long history of use in traditional medicine, with documented applications in China, Egypt, and India (Amjed et al. 2017). It exhibits a wide range of biological activities, such as antibacterial, anticancer, antioxidant, and anti-inflammatory effects (Gao et al. 2019). The primary bioactive constituents of *A. vera* include aloin and aloe emodin (AE) (Arsène et al. 2023). Our previous studies demonstrated that aloin significantly reduces the pathogenicity of *C. albicans* by remodeling the fungal cell wall and inducing the formation of abnormal hyphae (Liao et al. 2025). AE, a natural anthraquinone compound derived from *A. vera* and other plants, exhibits broad pharmacological activities (Wamer et al. 2003) including inhibition of tumor cell proliferation (Chen et al. 2007; Lee et al. 2010) and antibacterial effects against both Gram-positive and Gram-negative bacteria (Radha and Laxmipriya 2015). It also scavenges free radicals and mitigates oxidative stress–induced cellular damage (Liu et al. 2015). However, its effects on *C. albicans* virulence and pathogenicity remain poorly understood.

Iron, an essential trace element in eukaryotes, plays a crucial role in energy metabolism, oxygen transport, and DNA synthesis (Pijuan et al. 2023). In *C. albicans*, deletion of *SEF1*, a transcription factor that regulates iron uptake, significantly attenuates virulence in systemic infection models (Chen et al. 2011). Similarly, strains cultured under iron-depleted conditions or lacking *FTR1*, an iron permease, are unable to sustain hyphal growth (Luo et al. 2021), highlighting the importance of iron for hyphal development and pathogenicity. Disruption of iron homeostasis also leads to mitochondrial dysfunction, severely impairing aerobic respiration and energy metabolism. These defects subsequently affect hyphal morphogenesis, antifungal drug tolerance, and overall virulence (Xu et al. 2014). Given the central role of iron metabolism in fungal pathogenicity, targeting iron homeostasis represents a promising antifungal strategy. AE has been shown to chelate divalent iron ions and regulate cellular iron homeostasis by modulating the expression of genes involved in intracellular iron metabolism (He et al. 2023). These properties suggest that AE may serve as a novel agent for inhibiting fungal virulence by disrupting iron homeostasis.

In this study, we investigated the antifungal activity of AE and its underlying mechanisms, with a particular focus on hyphal development in *C. albicans*. We demonstrate, for the first time, that AE disrupts iron homeostasis, leading to mitochondrial dysfunction and impaired energy metabolism, which subsequently downregulate the cAMP–PKA signaling pathway and inhibit hyphal development and pathogenicity in oral candidiasis. These findings provide new insights into antifungal therapeutic targets and expand the potential applications of traditional herbal medicine in antifungal drug development.

## Materials and methods

### Chemicals

Aloin A (CAS: 1415-73-2, HPLC ≥ 98%), aloin B (CAS: 28371-16-6, HPLC ≥ 98%), AE (CAS: 481-72-1, HPLC ≥ 98%), acetyl coenzyme A (CAS: 102029-73-2, HPLC ≥ 93%), nystatin (CAS: 1400-61-9), amphotericin B (AmB; CAS: 1397-89-3), and fluconazole (FLC; CAS: 86386-73-4) were purchased from Solarbio (Beijing, China). *A. vera* extracts (CAS: 85507-69-3, HPLC ≥ 98%) were obtained from Wuhan Plange Biotechnology Co., Ltd. (Wuhan, China). Aloesin (CAS: 30861-27-9, HPLC ≥ 98%) was sourced from Yuanye Bio-Technology (Shanghai, China). 7-*O*-Methylaloeresin A (CAS: 329361-25-3, HPLC ≥ 98%) was procured from Desite Bio-Technology (Chengdu, China). Aloenin (CAS: 38412-46-3, HPLC ≥ 98%) and aloeresin D (CAS: 105317-67-7, HPLC ≥ 98%) were supplied by ChemFaces (Wuhan, China). Dimethyl sulfoxide (DMSO) was acquired from MP Biomedicals (Irvine, CA, USA). All compounds were dissolved in DMSO and stored at −20 °C.

### Strains and cells

*C. albicans* SC5314 (ATCC MYA-2876), clinical isolates (CCCC-2217, -2220, -2221, -2223, -2230, -2240, -2243, -2291, -2293, -2347, and -2349), and mutant strains (*ras1Δ/Δ*, *cyr1Δ/Δ*, *tpk1Δ/Δ*, and *tpk2Δ/Δ*) were obtained from the State Key Laboratory of Oral Diseases. The *RAS1*- and succinate dehydrogenase 2 (*SDH2*)-overexpressing strains (*RAS1 OE* and *SDH2 OE*) were constructed in this study. All strains are listed in Supplementary Table S1. All strains were cultured overnight at 35 °C in YPD medium. Human oral keratinocyte (HOK) cells (ScienCell, Carlsbad, CA, USA) and RAW264.7 macrophage cells (ATCC, Manassas, VA, USA) were maintained in DMEM medium.

### Hyphal formation measurement

*C. albicans* cells were suspended at 2 × 10^4^ colony-forming units (CFU)/mL in RPMI 1640 medium (Gibco, Scoresby, Victoria, Australia) and treated with 100 μg/mL of the eight compounds listed above or with DMSO (control, final concentration matched to that of treated groups and never exceeding 1 ‰) at 37 °C under 5% CO₂ for 24 h. Hyphal development was evaluated using a Cytation 5 imaging system (BioTek, Winooski, VT, USA).

Similarly, *C. albicans* cells were treated with 50, 100 μg/mL AE or DMSO for 5 h. Hyphal development was observed using a Leica E24HD stereomicroscope (Leica, Wetzlar, Germany) and quantitatively analyzed in ImageJ, with at least 200 cells per sample. Fungal suspensions were also spotted onto Spider agar plates containing AE to assess hyphal development over 5 days.

### Growth curve testing

*C. albicans* cells (2 × 10^6^ CFU/mL in YPD medium) were treated with nystatin (0, 0.75, and 1.5 μg/mL), AmB (0, 0.5, and 10 μg/mL), FLC (0, 5, and 10 μg/mL), AE (0, 50, and100 μg/mL), or DMSO. Fungal growth was monitored hourly for 24 h. The optical density at OD_600_ was measured using a SpectraMax ID3 microplate reader (SpectraMax ID3, Thermo Fisher Scientific, Waltham, MA, USA).

### Cytotoxicity assay

Cytotoxicity was assessed using a CCK-8 assay kit (Dojindo, Nara, Japan). The assay is based on the dehydrogenase-mediated reduction of a tetrazolium salt to a soluble yellow formazan dye. HOK cells (5 × 10^4^ cells/mL in DMEM) were treated with AE (0, 6.25, 12.5, 25, 50, and 100 μg/mL) for 24 h. Then, each well received 10 μL of CCK-8 reagent. After 2 h, the absorbance at 450 nm was read on a microplate reader.

### Cell adhesion assay

*C. albicans* cells (2 × 10^5^ CFU/mL in DMEM) were treated with 50 or 100 μg/mL AE—concentrations validated to inhibit hyphal formation without significant cytotoxicity (all subsequent experiments were performed using these concentrations)—or with DMSO. HOK cells were infected with the treated fungal suspensions for 1 h. After removal of non-adherent cells and washing, host cells were lysed with sterile water. The released fungal cells were plated on YPD agar plates for colony enumeration.

### Cell invasion assay

*C. albicans* cells (2 × 10^5^ CFU/mL in DMEM) were treated with 50 or 100 μg/mL AE and used to infect HOK cells for 3 h. Cells were then fixed overnight at 4 °C in 4% paraformaldehyde. Extracellular hyphae were stained with Alexa Fluor® 647–conjugated concanavalin A (ConA-647; Thermo Fisher Scientific, Waltham, MA, USA) for 45 min, followed by permeabilization with 0.1% Triton X-100 for 15 min. Invaded hyphae were stained with calcofluor white (Sigma-Aldrich, St. Louis, MO, USA) for 20 min. A confocal microscope (FV1000, Olympus, Tokyo, Japan) was used to image the samples, and invasion rates were calculated based on analysis of at least 100 fungal cells per sample.

### Cell damage assessment

*C. albicans* (1 × 10^5^ CFU/mL in DMEM) was treated with AE (50 or 100 μg/mL). After 24 h of co-culture with HOK cells in 96-well plates, host cell damage was assessed by measuring lactate dehydrogenase (LDH) activity in the culture supernatant using a microplate reader and an LDH detection kit (Roche, Basel, Switzerland).

### Susceptibility of *C. albicans* to macrophages

*C. albicans* cells (1 × 10^5^ CFU/mL in DMEM) treated with 50 or 100 μg/mL AE were added to overnight-cultured RAW264.7 macrophages (1 × 10^5^ cells/mL in DMEM) and incubated for 1 h. Cells were then lysed with sterile water, and the lysates were plated on YPD agar plates for colony counting.

### Measurement of the mitochondrial membrane potential (MMP)

*C. albicans* (1 × 10^7^ CFU/mL in DMEM) was treated with AE (50 or 100 μg/mL) or DMSO for 1 h. MMP was measured using a JC-1 assay kit (Solarbio, Beijing, China). After adding JC-1 staining solution (10 μg/mL) to each sample, incubation proceeded for 20 min. The cells were stained and analyzed by flow cytometry and fluorescence microscopy. For flow cytometric analysis, red fluorescence (indicative of high MMP) was detected at Ex 540 nm and Em 590 nm. In contrast, green fluorescence (indicative of low MMP) was detected at Ex 485 nm and Em 530 nm. For fluorescence microscopy, only red fluorescence was captured to visualize MMP distribution in fungal cells.

### ATP and cAMP extraction and measurement

*C. albicans* cells were treated with AE (50 or 100 μg/mL) or DMSO at 35 °C for 0, 0.5, 1 h, and overnight. Cells were harvested and lysed. Intracellular ATP and cAMP levels were quantified using commercial assay kits according to the manufacturer’s instructions. Luminescence and absorbance were measured using a microplate reader.

### Quantitative real-time polymerase chain reaction (qRT-PCR)

*C. albicans* (1 × 10^8^ CFU/mL) was treated with 100 μg/mL AE or DMSO for 2 h. Following collection, cells were resuspended in 1 mL TRIzol reagent and mechanically homogenized. Total RNA extraction was followed by reverse transcription into cDNA, and subsequent RT-PCR analysis was performed according to the provided protocol. Gene expression levels were measured by real-time PCR, using the primers detailed in Supplementary Table S2.

### Transcriptome sequencing and metabolomic analysis

*C. albicans* cells were treated with 100 μg/mL AE or DMSO for 6 and 12 h for transcriptomic analysis, and for 3 and 6 h for metabolomic analysis. Cells were collected and flash-frozen in liquid nitrogen. Total RNA extraction, transcriptome sequencing, and metabolomic analyses were performed by OE Biotech (Shanghai, China) and Shanghai Lu-Ming Biotech Co., Ltd. (Shanghai, China), respectively.

### Overexpression of *RAS1* and *SDH2* in *C. albicans*

The plasmid pJK-caCas9-NatMX-Neut5L (Addgene plasmid no. 89576) was obtained from Addgene (Watertown, MA, USA). The *CAS9* sequence in this plasmid was replaced with the *RAS1* or *SDH2* gene sequences. Gene synthesis and plasmid construction were performed by Sangon Biotech (Shanghai, China). The *RAS1* and *SDH2* overexpression plasmids were linearized using the *Pac*I restriction endonuclease (New England Biolabs, Ipswich, MA, USA) and transformed into *C. albicans* using the Fast Yeast Transformation™ Kit (G-Biosciences, St. Louis, MO, USA). qRT-PCR confirmed successful overexpression of *RAS1* and *SDH2*.

### Co-localization analysis

After treatment with 25 μg/mL AE (an optimal concentration for detecting its autofluorescence) or DMSO for 3 h, *C. albicans* cells were collected and stained with 2 nM MitoTracker Green (Beyotime Biotechnology, Shanghai, China) for 30 min. Following removal of excess dye, cells were observed using a confocal microscope, with green fluorescence indicating mitochondria and red autofluorescence indicating AE.

### Mitochondrial iron measurement

*C. albicans* suspensions (1 × 10^6^ CFU/mL) were treated overnight with AE (50 or 100 μg/mL) or DMSO. Cells were stained with 5 μM Mito-FerroGreen (Dojindo, Kumamoto, Japan) for 30 min, as previously described (Friedman et al. 2020). Fluorescence intensity was read on a microplate reader.

### SDH activity assay

*C. albicans* suspensions (5 × 10^6^ CFU/mL) were treated with AE (50 or 100 μg/mL) or DMSO. After 1 h, cells were collected, and SDH activity was measured using an SDH activity assay kit (Solarbio, Beijing, China).

### Murine model of oral candidiasis

Female BALB/c mice (4–6 weeks old) were obtained from DOSSY Co., Ltd. (Chengdu, China). *C. albicans* suspensions (1 × 10^7^ CFU/mL in saline) were prepared, and mice were anesthetized by intraperitoneal injection of tribromoethanol (20 μL/g body weight). Sublingual infection was established by placing a cotton ball saturated with the fungal suspension under the tongue for 75 min. Mice were provided with drinking water containing AE (50 or 100 μg/mL) and DMSO (control), or nystatin (100,000 IU/kg; positive control). Four days after infection, tongues were harvested for assessment of fungal burden and histopathological analysis. Each tongue was longitudinally divided into two parts: one for homogenization and fungal load quantification, and the other for histological analysis performed by Chengdu Aochuang Biotechnology Co., Ltd. (China).

### Statistical analysis

Data were analyzed in GraphPad Prism 10.1.2 (GraphPad Software, San Diego, CA, USA), with statistical significance set at *P-*value < 0.05. Parametric tests (*t*-test, one-way ANOVA, or Dunnett’s T3) or non-parametric tests (Kruskal–Wallis or Mann–Whitney *U*) were selected based on data distribution.

## Results

### AE inhibits hyphal development of *C. albicans*

**Fig. 1:**
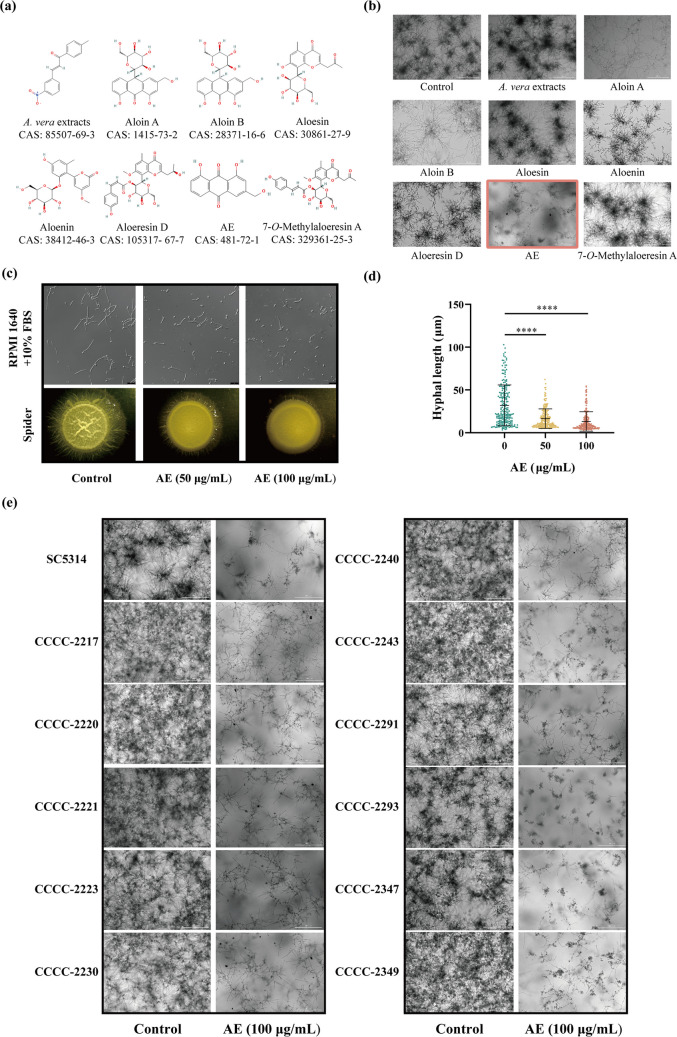
AE inhibits hyphal development of *C. albicans.* **a** Chemical structures of the eight aloe-derived monomeric compounds included in this study. **b** Effects of different aloe-derived monomeric compounds on hyphal formation in *C. albicans*. Cultures were grown in RPMI 1640 medium with 10% fetal bovine serum (FBS) overnight at 37 °C. **c** Live-cell imaging in RPMI 1640 medium with 10% FBS and Spider agar plate assays of *C. albicans* treated with 0, 50, and 100 μg/mL AE. **d** Quantitative analysis of hyphal length in *C. albicans* treated with 0, 50, and 100 μg/mL AE for 5 h. **e** Live-cell imaging of *C. albicans* SC5314 and clinical isolates treated with 0 and 100 μg/mL AE for 24 h. ****P < 0.0001

First, eight compounds derived from aloe were screened for their effects on *C. albicans* (Fig. 1a). In the control group, *C. albicans* formed abundant hyphae in RPMI 1640 medium, and *A. vera* extracts, aloesin, aloenin, aloeresin D, and 7-*O*-methylaloeresin A showed no significant effects on hyphal formation (Fig. 1b). In contrast, treatment with aloin A and aloin B resulted in thinner hyphae with fewer branches, while AE markedly inhibited hyphal formation in *C. albicans* (Fig. 1b). Unlike conventional antifungal drugs such as nystatin, FLC and AmB, which exhibit direct fungicidal activity (Supplemental Fig. S1a–c), AE showed no inhibitory effect on the growth of *C. albicans* (Supplemental Fig. S1d). Because the morphogenetic transition from yeast to hyphae is a critical virulence determinant of *C. albicans*, this pronounced inhibitory effect on hyphal development established AE as the lead compound for further investigation. Treatment with 50 and 100 μg/mL AE significantly inhibited *C. albicans* hyphal formation in both liquid medium and on solid plates (Fig. 1c). Hyphal length was correspondingly reduced (Fig. 1d). Furthermore, similar to its effect on the reference strain *C. albicans* SC5314, AE effectively suppressed hyphal formation in all 11 clinical isolates obtained from patients with oral candidiasis (Fig. 1e).

### AE inhibits the pathogenic effects of *C. albicans* on host cells

**Fig. 2:**
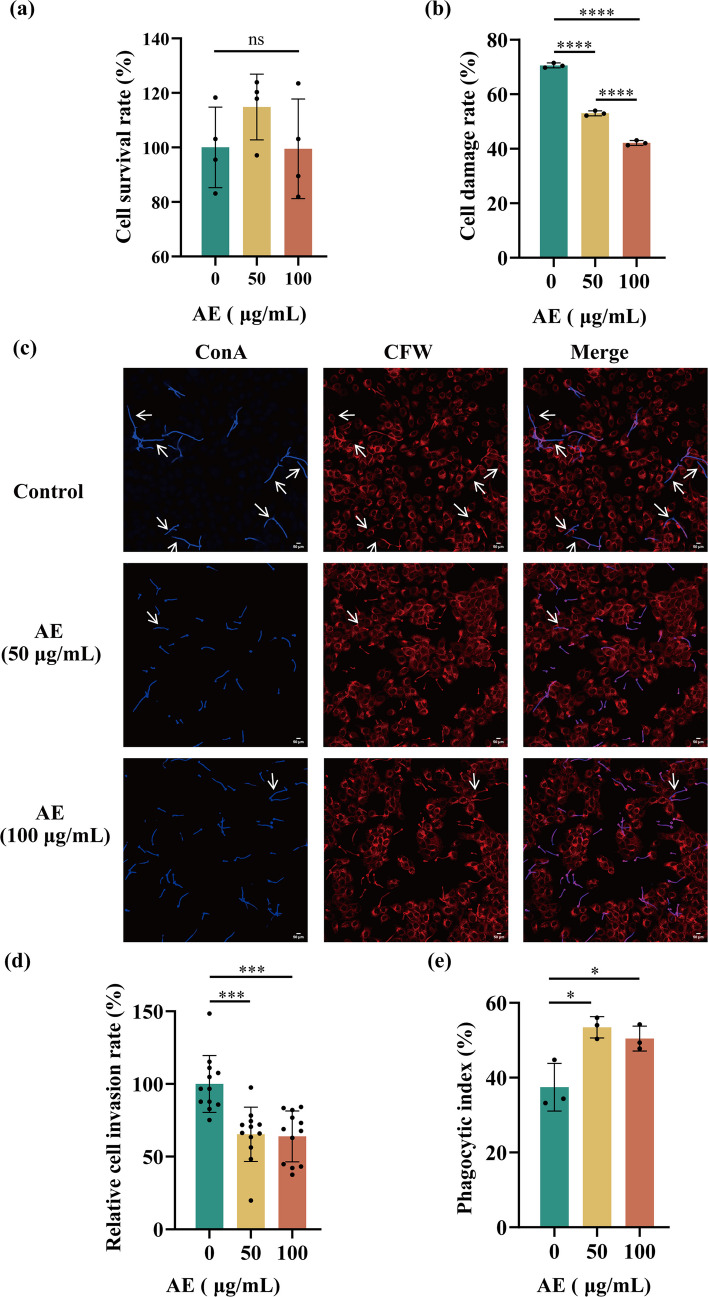
AE inhibits the pathogenic effects of *C. albicans* on host cells. **a** Cytotoxic effects of AE (0, 50, and 100 μg/mL) on HOK cells. **b** AE at 50 and 100 μg/mL reduced *C. albicans*–induced damage to HOK cells after 24 h of treatment. **c** Confocal images showing that 50 and 100 μg/mL AE reduced the invasive capacity of *C. albicans* in oral epithelial cells. Extracellular *C. albicans* was labeled with ConA-647. After membrane permeabilization, Calcofluor White was used to stain all fungal cells. Intracellular *C. albicans* hyphae are indicated by white arrows. **d** Quantification of *C. albicans* invasion into HOK cells after 3 h of treatment with 0, 50, and 100 μg/mL AE, expressed as the number of invasive hyphae. **e** AE at 50 and 100 μg/mL enhanced macrophage phagocytic efficiency against *C. albicans* after 1 h of treatment. ***P < 0.001; ****P < 0.0001; and ns, not significant

HOKs were then used to assess host–pathogen interactions. AE exhibited no cytotoxicity toward HOK cells at concentrations of 50 and 100 μg/mL (Fig. 2a). During *C. albicans* infection of HOK cells, AE significantly reduced host cell damage in a dose-dependent manner (Fig. 2b). Although AE did not affect fungal adhesion to HOK cells at 50 and 100 μg/mL (Supplemental Fig. S2), it significantly reduced fungal invasion (Fig. 2c–d). In addition, AE enhanced the phagocytic uptake of *C. albicans* by macrophages (Fig. 2e). Collectively, these findings demonstrate that AE suppresses *C. albicans* pathogenicity toward oral epithelial cells and limits immune evasion from macrophages by inhibiting hyphal development.

### AE remodels the energy metabolism of *C. albicans*

**Fig. 3:**
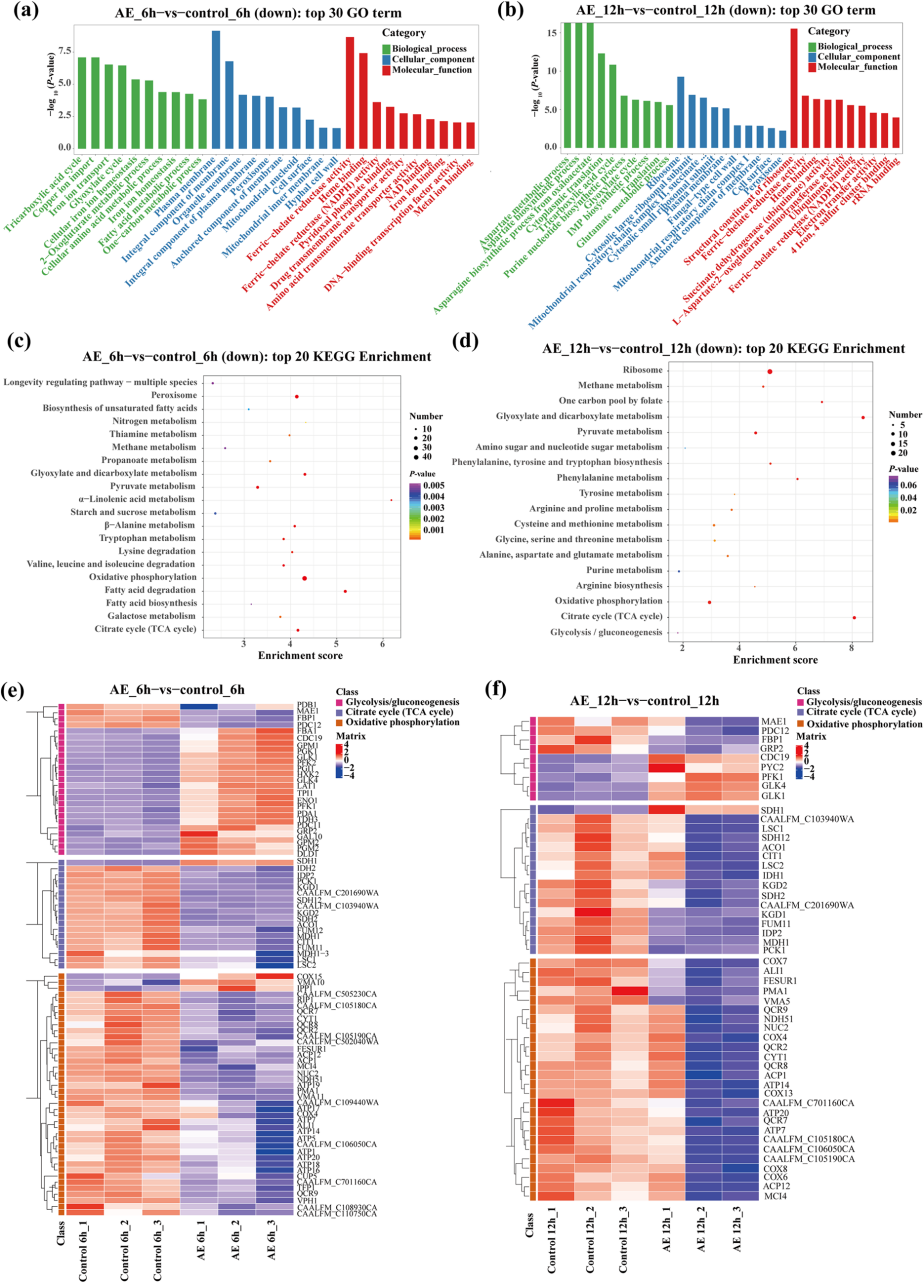
AE induces mitochondrial dysfunction of *C. albicans*. **a**, **b** Top 30 enriched GO terms in *C. albicans* following 6 h (**a**) and 12 h (**b**) treatment with 100 μg/mL AE. The horizontal and vertical axes represent GO terms and −log_10_(*P-*value), respectively. **c**, **d** Bubble plots of the top 20 significantly downregulated genes enriched in KEGG pathways after 6 h (**c**) and 12 h (**d**) treatment with 100 μg/mL AE. **e**, **f** Heatmaps of significantly altered genes involved in glycolysis/gluconeogenesis, the tricarboxylic acid cycle, and oxidative phosphorylation pathways after 6 h (**e**) and 12 h (**f**) treatment with 100 μg/mL AE. Sample groups and genes are shown on the horizontal and vertical axes, respectively

To investigate the mechanism underlying AE-mediated inhibition of hyphal development, *C. albicans* was treated with 100 μg/mL AE for 6 and 12 h, followed by transcriptomic sequencing and analysis. Gene Ontology (GO) and Kyoto Encyclopedia of Genes and Genomes (KEGG) analyses of differentially expressed genes revealed significant enrichment of pathways related to energy metabolism, carbohydrate metabolism, and mitochondrial function (Fig. 3a–d). These transcriptional changes prompted the hypothesis that AE disrupts mitochondrial energy metabolism. Further analysis of metabolism-related genes revealed that AE significantly upregulated genes involved in glycolysis/gluconeogenesis while downregulated genes associated with the tricarboxylic acid (TCA) cycle and oxidative phosphorylation (Fig. 3e–f). This transcriptional shift is consistent with metabolic remodeling away from efficient, mitochondria-dependent oxidative phosphorylation toward the less efficient glycolytic pathway (Czyz et al. 2017), further suggesting that AE impairs mitochondrial function and induces metabolic reprogramming to inhibit hyphal development. Because mitochondrial function is essential for hyphal morphogenesis, we propose that AE-induced mitochondrial dysfunction and the resulting metabolic remodeling compromise cellular energy production, thereby suppressing hyphal development.

**Fig. 4:**
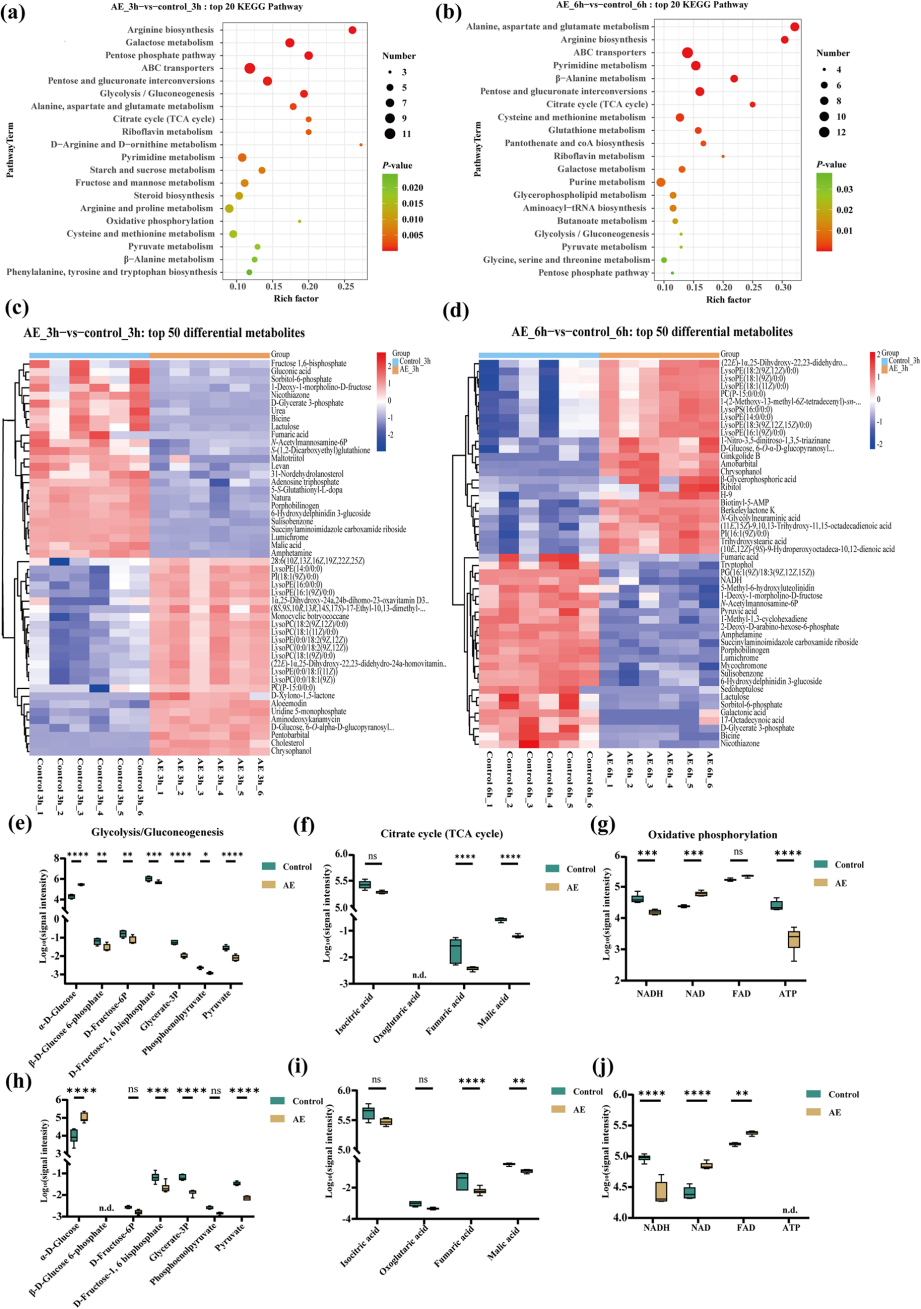
AE remodels the energy metabolism in *C. albicans*. **a**, **b** Bubble plots of the top 20 significantly altered metabolic pathways enriched in KEGG analysis after 3 h (**a**) and 6 h (**b**) treatment with 100 μg/mL AE. **c**, **d** Heatmaps of the top 50 significantly altered metabolites after 3 h (**c**) and 6 h (**d**) treatment with 100 μg/mL AE. Sample groups and metabolites are shown on the horizontal and vertical axes, respectively. **e**–**g** Changes in the levels of key metabolites involved in glycolysis (**e**), the tricarboxylic acid (TCA) cycle (**f**), and oxidative phosphorylation (**g**) in *C. albicans* treated with 0 and 100 μg/mL AE for 3 h. **h**–**j** Changes in the levels of key metabolites involved in glycolysis (**h**), the TCA cycle (**i**), and oxidative phosphorylation (**j**) in *C. albicans* treated with 0 and 100 μg/mL AE for 6 h

To further validate the transcriptomic findings and confirm AE-induced metabolic remodeling, metabolomic analyses of *C. albicans* were performed. Consistent with the transcriptomic data, pathways related to energy metabolism—including glycolysis/gluconeogenesis, the TCA cycle, and oxidative phosphorylation—were significantly enriched (Fig. 4a–b). Metabolomic analysis revealed that after 3 h of AE treatment, levels of key glycolytic intermediates, including glucose-6-phosphate, fructose-6-phosphate, fructose-1,6-bisphosphate, glycerate-3-phosphate, phosphoenolpyruvate, and pyruvate, were significantly reduced. At the same time, the upstream metabolite α-D-glucose was significantly accumulated (Fig. 4c, e). Concurrently, levels of TCA cycle intermediates, such as fumaric acid and malic acid (Fig. 4f), as well as oxidative phosphorylation components, including NADH and ATP (Fig. 4g), were markedly decreased. Similar metabolite alterations persisted after 6 h of AE treatment (Fig. 4d, h–j), providing strong multi-omics evidence for AE-induced metabolic remodeling in *C. albicans*. Given the central role of acetyl coenzyme A (acetyl-CoA) as the initiating substrate and a key metabolic hub in the TCA cycle (Arnold and Finley 2023), exogenous acetyl-CoA was added to assess its effect on AE activity. Acetyl-CoA partially reversed the inhibitory effect of AE on hyphal development (Supplemental Fig. S3), further supporting the conclusion that AE suppresses hyphal development by remodeling *C. albicans* energy metabolism.

### AE downregulates the cAMP–PKA pathway to inhibit *C. albicans* hyphal development

**Fig. 5:**
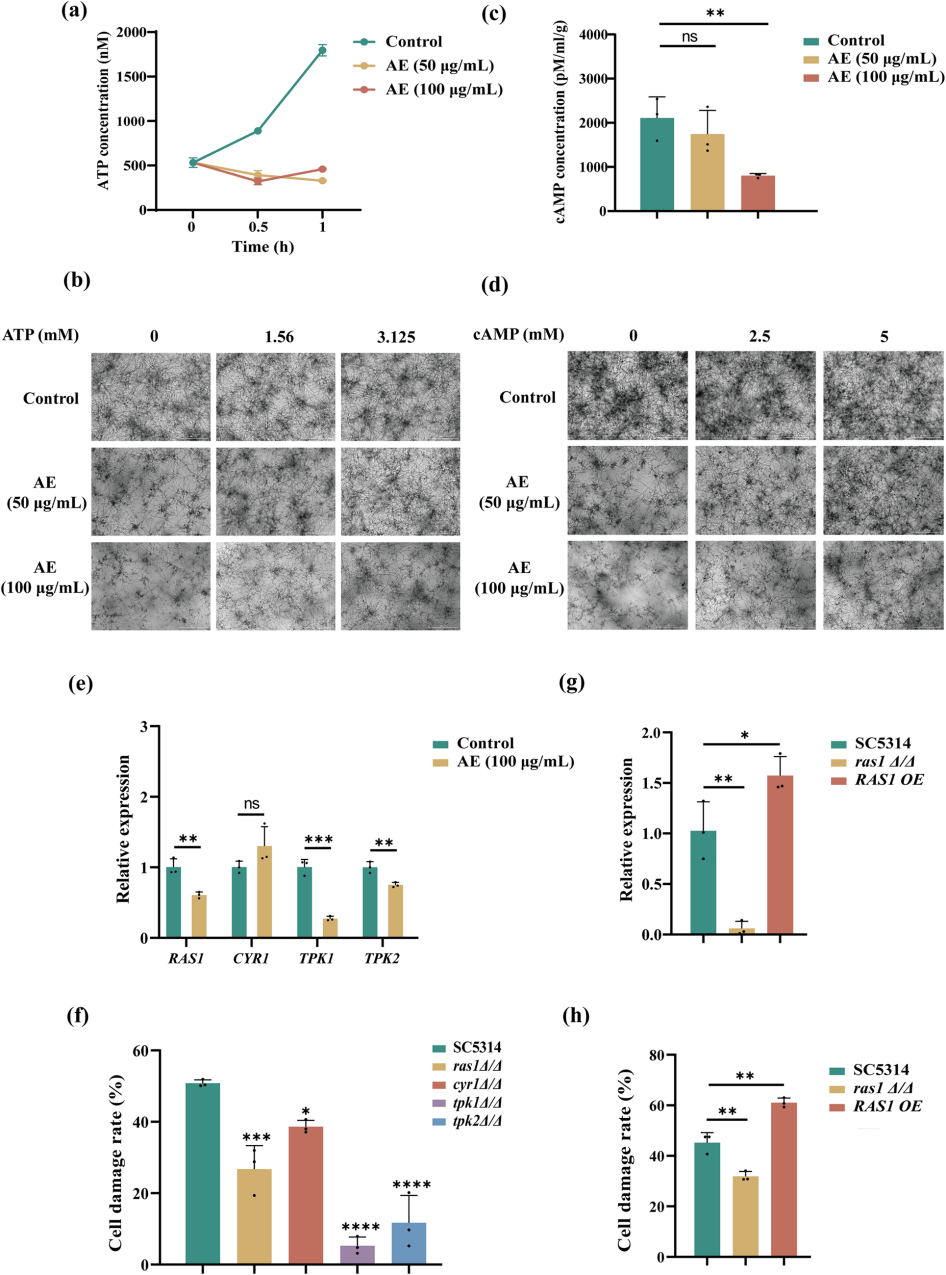
AE downregulates the cAMP-PKA pathway to inhibit *C. albicans* hyphal development. **a**–**d** Relative intracellular ATP (**a**) and cAMP (**c**) levels in *C. albicans* following treatment with 0, 50, and 100 μg/mL AE. Live-cell imaging shows restoration of hyphal growth by exogenous ATP (**b**) and cAMP (**d**) under AE treatment. **e** Expression of cAMP–PKA pathway genes following treatment with 100 μg/mL AE for 2 h. **f** Null mutants enhanced the inhibitory effect of 100 μg/mL AE on *C. albicans*–induced damage to human oral keratinocyte cells*.* **g** Expression levels of *RAS1* in the SC5314, *ras1Δ/Δ*, and *RAS1 OE* strains. **h** The inhibitory effect of 100 μg/mL AE on *C. albicans*–induced epithelial cell damage was enhanced in the *ras1Δ/Δ* mutant but attenuated in the *RAS1 OE* strain. *P < 0.05; **P < 0.01; ***P < 0.001; ****P < 0.0001; and ns, not significant

To further explore the mechanisms by which AE inhibits *C. albicans* hyphal development through metabolic remodeling, we hypothesized that AE downregulates the cAMP–PKA signaling pathway, which is known to regulate hyphal development under conditions of altered metabolism in *C. albicans* (Chen et al. 2020a; Lassak et al. 2011). Intracellular ATP levels in *C. albicans* were first measured. AE treatment significantly reduced ATP production (Fig. 5a). Notably, supplementation with exogenous ATP reversed the inhibitory effect of AE on hyphal formation (Fig. 5b). AE also significantly decreased intracellular cAMP levels (Fig. 5c), consistent with the reduction in ATP production, and exogenous cAMP similarly reversed AE-mediated inhibition of hyphal development (Fig. 5d). These results indicate that AE remodels *C. albicans* metabolism, leading to reduced ATP and cAMP production and, consequently, impaired hyphal formation. Given the reduction in cAMP levels, the expression of key genes in the cAMP–PKA signaling pathway was assessed by qRT-PCR. AE significantly downregulated the expression of *RAS1*, *TPK1*, and *TPK2* (Fig. 5e). To further validate the involvement of this pathway, null mutants (*ras1Δ/Δ*, *cyr1Δ/Δ*, *tpk1Δ/Δ*, and *tpk2Δ/Δ*) were examined for their ability to induce damage in oral epithelial cells. Deletion of *RAS1*, *CYR1*, *TPK1*, or *TPK2* enhanced the inhibitory effects of AE on epithelial cell damage (Fig. 5f). In contrast, in the *RAS1* overexpression strain (*RAS1 OE*) (Fig. 5g), the inhibitory effect of AE on epithelial cell damage was significantly attenuated (Fig. 5h). Importantly, AE did not affect the growth of these mutant or overexpression strains (Supplemental Fig. S4a–e). Collectively, these results demonstrate that AE inhibits *C. albicans* hyphal development and pathogenicity by downregulating the cAMP–PKA signaling pathway as a consequence of metabolic reprogramming and reduced intracellular ATP and cAMP levels.

### AE disrupts mitochondrial iron homeostasis and reduces succinate dehydrogenase 2 (SDH2) activity

**Fig. 6:**
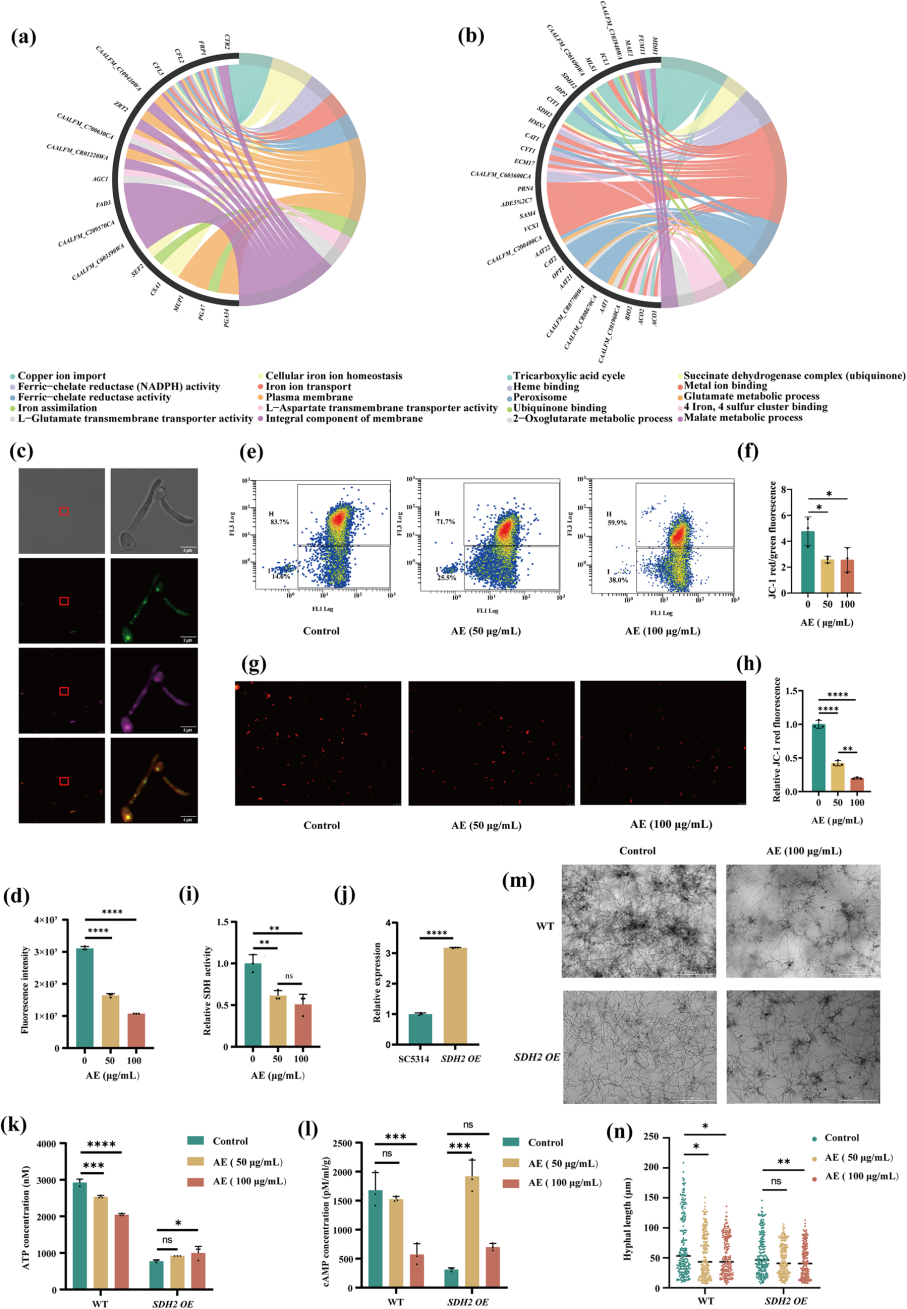
AE disrupts mitochondrial iron homeostasis and reduces succinate dehydrogenase 2 (SDH2) activity. **a**, **b** GO enrichment analysis of the green-yellow (**a**) and blue (**b**) weighted gene co-expression network analysis modules containing downregulated genes in *C. albicans* following treatment with 100 μg/mL AE. **c** Confocal microscopy showing colocalization of AE with mitochondria. Mitochondria are shown in green, AE autofluorescence in magenta, and overlapping signals are shown in the merged images. **d** Mitochondrial iron content in *C. albicans* following treatment with 0, 50, or 100 μg/mL AE, expressed as fluorescence intensity. **e** MMP of *C. albicans* assessed by JC-1 staining and flow cytometry, with red and green fluorescence indicating JC-1 aggregates and monomers, respectively. **f** Quantification of MMP based on the red-to-green fluorescence ratio. **g** Fluorescence microscopy images of *C. albicans* treated with 0, 50, and 100 μg/mL AE; red puncta indicate functional mitochondria. **h** Quantitative analysis of mitochondrial fluorescence intensity measured using a microplate reader. **i** SDH activity in *C. albicans* following treatment with 0, 50, and 100 μg/mL AE. **j** Expression levels of *SDH2* in SC5314 and *SDH2 OE* strains. **k**, **l** Relative intracellular ATP (**k**) and cAMP (**l**) levels in wild-type (WT) and *SDH2 OE* strains following treatment with 0, 50, and 100 μg/mL AE. **m** Live-cell imaging of WT and *SDH2 OE* strains treated with AE for 24 h. **n** Quantitative analysis of hyphal length in WT and *SDH2 OE* strains treated with 0, 50, and 100 μg/mL AE. *P < 0.05; **P < 0.01; ***P < 0.001; ****P < 0.0001; and ns, not significant

To further elucidate how AE reprograms *C. albicans* metabolism, weighted gene co-expression network analysis (WGCNA) integrating transcriptomic and metabolomic datasets was performed. Significantly downregulated genes were enriched in two modules, designated green-yellow and blue (Supplemental Fig. S5). GO enrichment analysis revealed that the green-yellow module was primarily associated with iron ion uptake and membrane transport processes, including ferric-chelate reductase activity, iron assimilation, and iron ion transport, all of which were significantly downregulated (Fig. 6a; Supplemental Fig. S6a). In contrast, the blue module was enriched for pathways related to the TCA cycle and mitochondrial energy metabolism, with a notable reduction in SDH activity (Fig. 6b; Supplemental Fig. S6b). SDH is a key enzyme that links the TCA cycle and the mitochondrial respiratory chain and is closely associated with cellular ATP production (Adolph et al. 2022). Collectively, the combined transcriptomic, metabolomic, and WGCNA analyses indicate that AE strongly impairs mitochondrial electron transport chain function, thereby reducing ATP production.

The effects of AE on mitochondrial function were subsequently examined. Confocal imaging revealed clear colocalization of AE with mitochondria in *C. albicans* cells (Fig. 6c). Importantly, AE significantly reduced mitochondrial iron content in a dose-dependent manner (Fig. 6d). Concurrently, AE markedly decreased the MMP of *C. albicans* (Fig. 6e–h), indicating that disruption of mitochondrial iron homeostasis by AE leads to mitochondrial dysfunction.

Consistent with transcriptomic and metabolomic analyses indicating impaired SDH activity, AE significantly reduced SDH activity in a dose-dependent manner (Fig. 6i). These findings suggest that AE disrupts mitochondrial iron homeostasis, thereby inhibiting SDH activity. To further validate this mechanism, an *SDH2* overexpression strain (*SDH2 OE*) was constructed. Overexpression of *SDH2* partially restored ATP production and intracellular cAMP levels in AE-treated *C. albicans* cells (Fig. 6j–l). Moreover, compared with the wild-type strain, the *SDH2 OE* strain exhibited attenuated sensitivity to AE-mediated inhibition of hyphal development (Fig. 6m–n). Together, these results further demonstrate that AE suppresses hyphal development by reducing SDH activity, reprogramming energy metabolism, and impairing mitochondrial function in *C. albicans*.

### AE inhibits oral candidiasis in mice

**Fig. 7:**
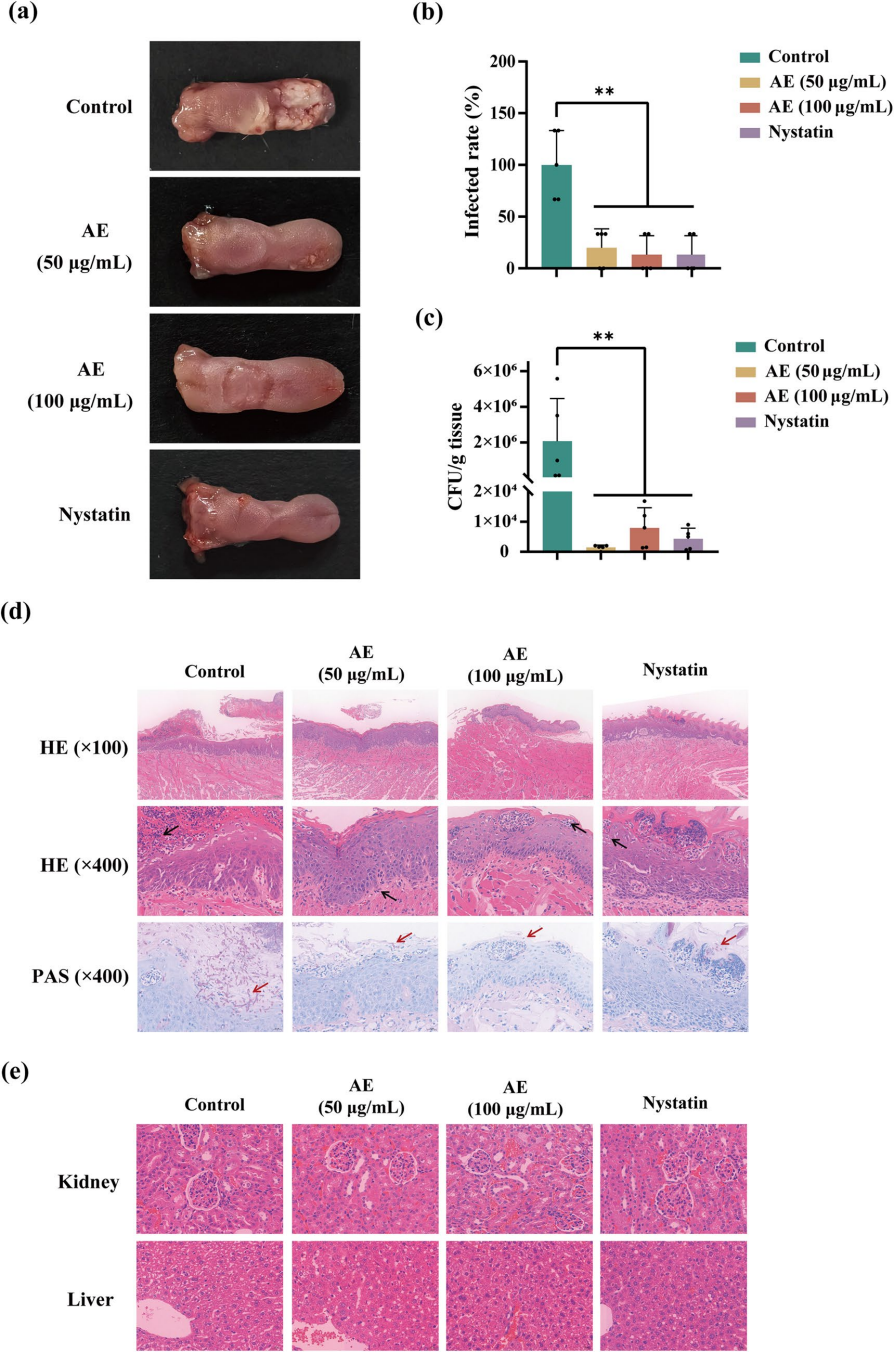
AE inhibits oral candidiasis in mice. **a** Representative oral phenotypes of mouse tongues 4 days after *C. albicans* infection following treatment with AE (0, 50, and 100 μg/mL) or nystatin (100,000 IU/kg). **b** Quantification of the epithelial area infected following treatment with AE and nystatin. **c** Fungal burden in mouse tongues 4 days after *C. albicans* infection. **d** HE (100× and 400× magnification) and PAS staining of mouse tongues 4 days post-infection following treatment with AE or nystatin. Black and red arrows indicate inflammatory cells and invasive hyphae, respectively. **e** HE staining (400× magnification) of kidney and liver tissues to evaluate systemic toxicity following administration of AE or nystatin via drinking water. **P < 0.01

Based on the pronounced inhibitory effects of AE on hyphal development in vitro and its lack of cytotoxicity, we next evaluated its antifungal efficacy in an in vivo mouse model of oral candidiasis. In the control group, the tongue mucosa was covered with a layer of white plaques, whereas treatment with 50 or 100 μg/mL AE markedly reduced plaque formation, with effects comparable to those observed in the nystatin-treated group (Fig. 7a–b). AE treatment also significantly reduced the fungal burden, again to a level comparable to that achieved with nystatin (Fig. 7c). Hematoxylin–eosin (H&E) and periodic acid–Schiff (PAS) staining demonstrated that AE markedly reduced inflammatory cell infiltration and hyphal invasion of *C. albicans* compared with the control group (Fig. 7d). Furthermore, HE staining of major metabolic organs, including the liver and kidneys, revealed no obvious histopathological abnormalities following AE treatment (Fig. 7e), indicating a lack of significant systemic toxicity. Collectively, these results demonstrate that AE exerts potent antifungal activity against oral candidiasis in vivo by inhibiting hyphal development.

## Discussion

The morphological transition of *C. albicans* is a critical virulence factor (Sudbery 2011). Suppression of the yeast-to-hypha transition can mitigate *C. albicans* pathogenicity, as this morphological shift is essential for host cell adhesion, invasion, tissue damage, and evasion of macrophage-mediated immune responses (Jacobsen et al. 2014). Aloe extracts have been reported to inhibit the growth, hyphal development, and biofilm formation of *C. albicans* (Gao et al. 2019; Liao et al. 2025). However, the specific antifungal components and their underlying mechanisms remain unclear. In this study, we screened eight commercially available monomeric aloe compounds for antifungal activity. Our results demonstrate, for the first time, that AE significantly suppresses hyphal formation and pathogenicity in *C. albicans* without directly affecting fungal viability, indicating that AE is a promising antivirulence agent against *C. albicans*.

Mitochondria are fundamental for maintaining normal cellular functions (Thomas et al. 2013). Confocal fluorescence imaging revealed colocalization of AE with mitochondria, and AE treatment reduced the MMP of *C. albicans*, indicating that AE impairs mitochondrial function and disrupts fungal energy and carbohydrate metabolism. This mitochondrial dysfunction likely represents an initiating event that reduces ATP production. The resulting energy deficiency subsequently suppresses the cyclic adenosine monophosphate–protein kinase A (cAMP–PKA) signaling pathway, a central regulator of hyphal development in *C. albicans* (Chen et al. 2020a). We observed that AE significantly downregulated intracellular cAMP levels, accompanied by marked downregulation of key pathway components, including *RAS1*, *TPK1*, and *TPK2*. Importantly, mutant strains (*ras1Δ/Δ*, *cyr1Δ/Δ*, *tpk1Δ/Δ*, and *tpk2Δ/Δ*) exhibited enhanced sensitivity to AE, whereas *RAS1* overexpression partially attenuated the inhibitory effect of AE on *C. albicans*–induced host cell damage. Together, these results indicate that AE impairs mitochondrial function, triggers metabolic remodeling, and subsequently inhibits the cAMP–PKA pathway, ultimately suppressing hyphal development in *C. albicans*.

Iron ions are essential for the survival, pathogenicity, and environmental adaptation of *C. albicans* (Monroy-Pérez et al. 2020). They regulate hyphal development and morphological transitions by modulating key transcription factors, including *Sef1*, *Hap43*, and *Sfu1* (Noble 2013; Singh et al. 2011). Iron serves as a critical cofactor for the biosynthesis of heme and iron–sulfur clusters (Lill and Freibert 2020). In this study, AE dose-dependently reduced mitochondrial iron levels in *C. albicans*, thereby disrupting iron homeostasis, likely owing to its reported ability to chelate iron ions and regulate the expression of genes involved in intracellular iron metabolism (He et al. 2023). Nevertheless, we cannot exclude the possibility that iron dyshomeostasis is also exacerbated as a secondary consequence of mitochondrial dysfunction, given the close interdependence of these processes (Fang et al. 2023). Iron ions are indispensable cofactors for numerous enzymes that drive essential biochemical processes, including respiration, DNA replication, and metabolic pathways (Pijuan et al. 2023). SDH, also known as mitochondrial complex II, represents a key metabolic node linking the TCA cycle to the electron transport chain (Pozza et al. 2020), and its activity depends on iron–sulfur clusters. Our metabolomic analysis revealed that AE significantly reduced SDH activity, thereby decreasing ATP and cAMP synthesis. In *SDH2* overexpression strains, these inhibitory effects were partially reversed. Collectively, these findings suggest that AE disrupts iron homeostasis and inhibits SDH activity, thereby reprogramming energy metabolism in *C. albicans*. Given the tight coupling between mitochondrial function and cellular metabolism, inhibition of hyphal development likely arises from the combined effects of this pathway and additional cellular targets of AE. As a natural compound with diverse bioactivities, including antibacterial and antiproliferative effects (Lee et al. 2010; Radha and Laxmipriya 2015), AE is likely to influence multiple interconnected nodes within fungal metabolic and signaling networks. Thus, the proposed model highlights a central mechanism underlying AE activity, although further studies are required to fully elucidate the integrated regulatory pathways involved.

This study also provides the first in vivo evaluation of the therapeutic efficacy of AE against oral candidiasis. AE treatment significantly reduced the area of tongue infection, fungal burden, tissue invasion, histopathological damage, and inflammatory responses compared with the control group, with effects comparable to those observed in the nystatin-treated group. These findings support AE as a potential antivirulence agent for the treatment of fungal infections. However, further studies are needed to comprehensively assess its safety, pharmacokinetics, and clinical applicability.

In summary, this study demonstrates, for the first time, that AE disrupts mitochondrial iron homeostasis in *C. albicans*, leading to mitochondrial dysfunction and metabolic remodeling, partially by inhibiting SDH. This disruption results in decreased intracellular ATP and cAMP levels, subsequent downregulation of the cAMP–PKA signaling pathway, and suppression of hyphal development and pathogenicity. These findings broaden the potential applications of AE and provide valuable insights into novel antifungal targets.

## Supplementary Information

Below is the link to the electronic supplementary material.

Supplementary file1 (PDF 1519 KB)

## Data Availability

The transcriptome sequencing and metabolome data were accessible online (https://dataview.ncbi.nlm.nih.gov/object/PRJNA1346170?reviewer=fup2jhu3pc6du4453gvr825b8s) and (10.21228/M8PC18), respectively.
