# Supplementary material for: Aloe emodin disrupts Candida albicans mitochondrial iron homeostasis against its hyphal development and oral candidiasis
Source: Appl Microbiol Biotechnol. 2026 Feb 20;110(1):71. doi: 10.1007/s00253-026-13740-1 (PMC12923502; doi:10.1007/s00253-026-13740-1)
Supplement: Supplementary file 1 — Supplementary file1 (PDF 1519 KB) [file 253_2026_13740_MOESM1_ESM.pdf]

Applied Microbiology and Biotechnology

Aloe emodin disrupts *Candida albicans* mitochondrial iron homeostasis against its hyphal development and oral candidiasis

Jiawei Shen [1, #], Chuanli Zhang [2, #], Yifan Lin [1], Chunfei Zhang [3], Jingzhi Zhou [1], Yujie Zhou [4], Lichen Gou [1], Ga Liao [1], Zhuoli Zhu [1, 5], Lei Cheng [1, 6], Binyou Liao [1, 7, *], Biao Ren [1, 8, *]

[1] State Key Laboratory of Oral Diseases, National Center for Stomatology, National Clinical Research Center for Oral Diseases, West China School of Stomatology, Sichuan University, Chengdu 610041, Sichuan, China

[2] Department of Hematology, West China Hospital, Sichuan University, Chengdu 610041, Sichuan, China

[3] School of nursing, Beijing University of Chinese medicine, Beijing 100029, China

[4] Hospital of Stomatology, Guangdong Provincial Key Laboratory of Stomatology, Guanghua School of Stomatology, Sun Yat-sen University, Guangzhou, 510055, Guangdong, China

[5] Department of Geriatric Dentistry, West China Hospital of Stomatology, Sichuan University, Chengdu 610041, Sichuan, China

[6] Department of Operative Dentistry and Endodontics, West China School of Stomatology, Sichuan University, Chengdu 610041, Sichuan, China

[7] Department of Periodontics, West China School of Stomatology, Sichuan University, Chengdu, 610041, Sichuan, China

[8] Tianfu Jiangxi Laboratory, Chengdu 641419, Sichuan, China

# Jiawei Shen and Chuanli Zhang contributed equally to this work. .

* To whom correspondence should be addressed:

Binyou Liao, liaobinyou@126.com

Biao Ren, renbiao@scu.edu.cn: ORCID: https://orcid.org/0000-0003-4215-2873

Supplementary figures and tables

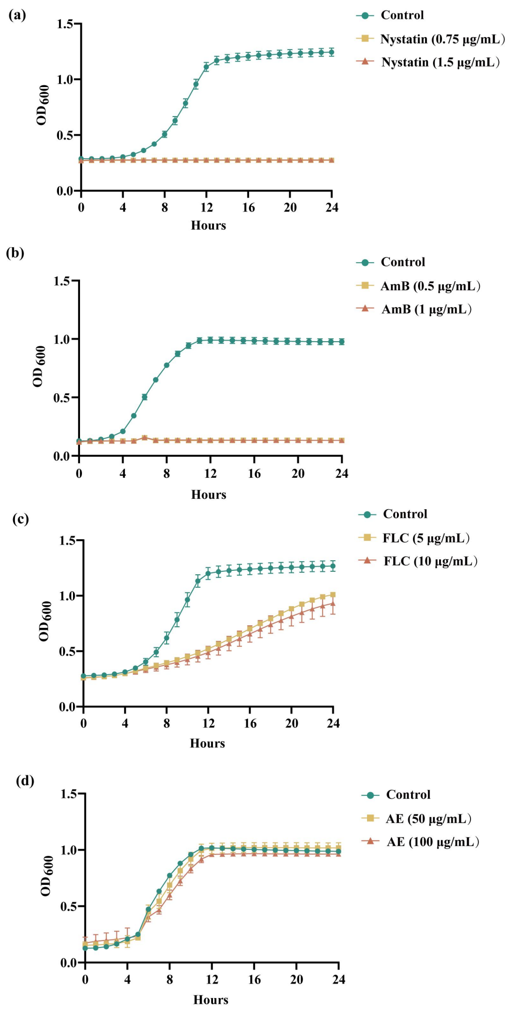

Fig. S1. Growth curve of *C. albicans*. The growth of *C. albicans* was monitored over 24 h in YPD medium containing 0, 0.75, 1.5 μg/mL nystatin (a), 0, 0.5, 1 μg/mL AmB (b), 0, 5, 10 μg/mL FLC (c), and 0, 50, 100 μg/mL AE (d) with OD_{600} measured at 1 h intervals.

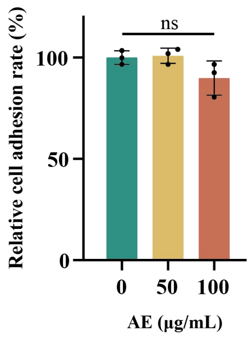

Fig. S2. Cellular adhesion of *C. albicans*. Adhesion rates of *C. albicans* to HOK cells after 1 h treatment with 0, 50, 100 μg/mL AE. ns, no significant difference.

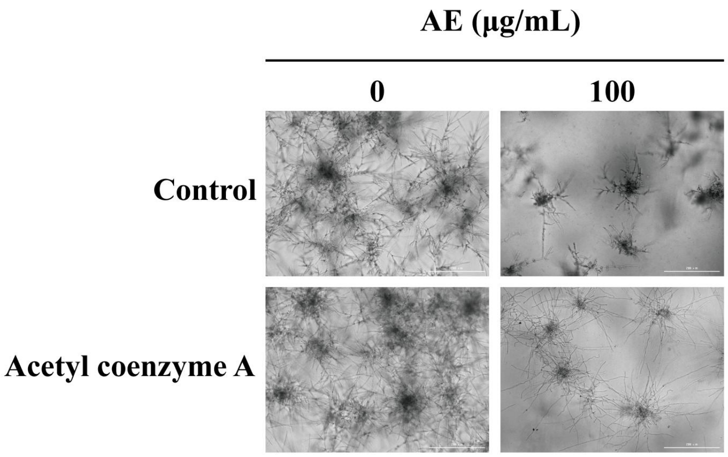

Fig. S3. Exogenous addition of acetyl coenzyme A reversed the hyphal inhibitory effect of aloe emodin. Live cell imaging showed that 5 mM exogenous Acetyl-CoA restored hyphal growth in *C. albicans* treated with 100 µg/mL AE.

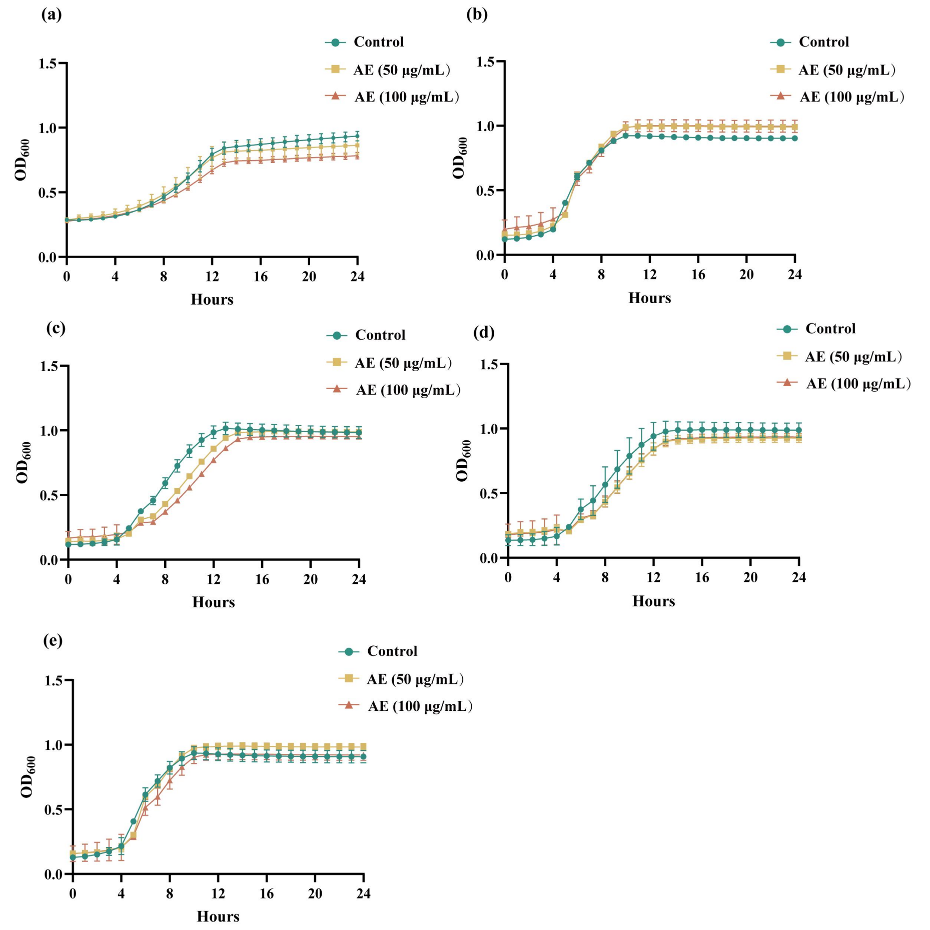

Fig. S4. Growth curves of *C. albicans* mutant and overexpression strains. The growth of *ras1Δ/Δ* (a), *cyr1Δ/Δ* (b), *tpk1Δ/Δ* (c), *tpk2Δ/Δ* (d), and *RAS1* overexpression strain (e) were monitored over 24 h in YPD medium containing 0, 50, 100 μg/mL AE with OD_{600} measured at 1 h intervals.

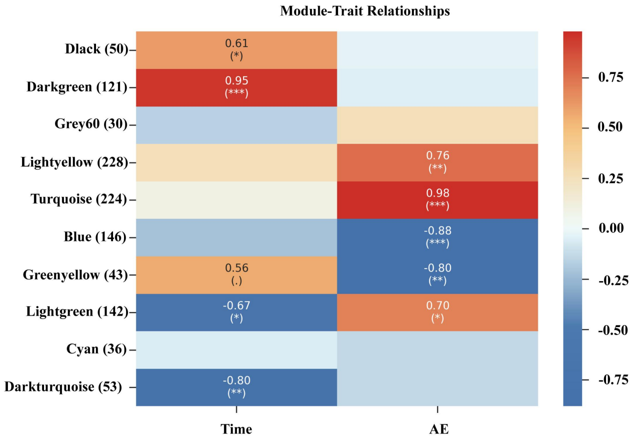

Fig. S5. The relationship of 2 traits and 11 modules. WGCNA of the transcriptomic and metabolomic data from *C. albicans* treated with 100 μg/mL AE identified 11 distinct co-expression modules.

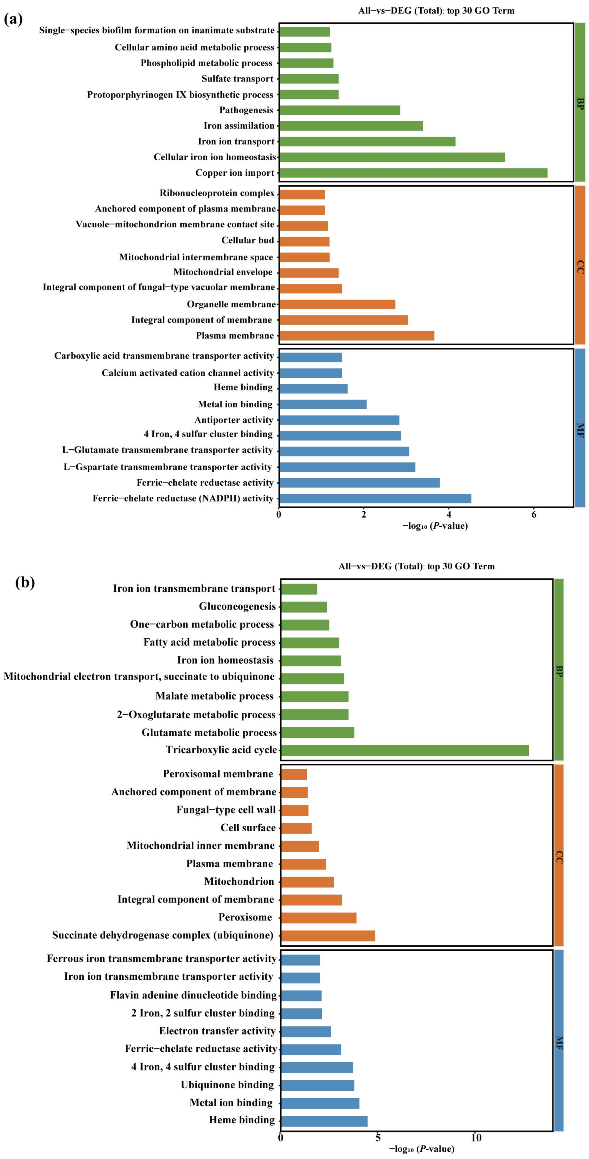

Fig. S6. Top 30 enriched GO terms for downregulated genes from WGCNA modules in *C. albicans* treated with 100 µg/mL aloe emodin. (a) The greenyellow module was significantly enriched in biological processes and cellular components related to iron ion homeostasis. (b) The blue module was predominantly enriched for mitochondrial and peroxisomal metabolic functions. The horizontal and vertical axes represented the GO terms and the -$\log_{10}$ (*P*-value), respectively.

Table S1. *Candida albicans* strains used in this study.

Stain	Genotype	Reference
SC5314	Parent strain, ATCC MYA - 2876	(Gillum et al. 1984)
ras1Δ/Δ	*ras1::hisG/ras1::hphURA3-hph*<br>*ura3::λimm434/ura3::λimm-434*	(Feng et al. 1999)
cyr1Δ/Δ	*cdc35::hisG/cdc35::hisG-URA3-hisG*<br>*ura3::λimm434/ura3::λimm-434*	(Mallet et al. 2000)
tpk1Δ/Δ	*tpk1::hisG/tpk1::hisG-URA3-hisG*<br>*ura3::λimm434/ura3::λimm434*	(Bockmühl et al. 2001)
tpk2Δ/Δ	*tpk2::hisG/tpk2::hisG-URA3-hisG*<br>*ura3::λimm434/ura3::λimm434*	(Bockmühl et al. 2001)
RAS1 OE	SC5314 *NEUT5L/NEUT5L-ACT1*<br>*promoter-Ras1-NAT1-Neut5L*	This study
SDH2 OE	SC5314 *NEUT5L/NEUT5L-ACT1*<br>*promoter-Sdh2-NAT1-Neut5L*	This study
CCCC-2217	Clinical isolate	This study
CCCC-2220	Clinical isolate	This study
CCCC-2221	Clinical isolate	This study
CCCC-2223	Clinical isolate	This study
CCCC-2230	Clinical isolate	This study
CCCC-2240	Clinical isolate	This study
CCCC-2243	Clinical isolate	This study
CCCC-2291	Clinical isolate	This study
CCCC-2293	Clinical isolate	This study
CCCC-2347	Clinical isolate	This study
CCCC-2349	Clinical isolate	This study

Table S2. Real-time PCR primers used in this study.

Primers		Nucleotide Sequence (5'-3')	Reference
ACT1	Forward sequence	TGGAAGCTGCTGGTATTGAC	(Zhu et al. 2021)
	Reverse sequence	TCCTTTTGCATACGTTCAGC	
RAS1	Forward sequence	GGTGGTGTTGGTAAATCCGCT	This study
	Reverse sequence	GGCCAGATATTCTTCTTGTCCAGC	
CYR1	Forward sequence	GCCCCTGAAAGTTGGGATGT	This study
	Reverse sequence	GTAACATGCGGCACTTGGTG	
TPK1	Forward sequence	GCTGCCGAAGTATTTTTGGCT	This study
	Reverse sequence	TGTTGCCACCACTTCAGGAG	
TPK2	Forward sequence	CTGGGGTTGGTGATTCGTCA	This study
	Reverse sequence	TCTCCTTGGCTTCCGTAGTC	
SDH2	Forward sequence	TCTTGTGTGCTTGTTGTTCT	This study
	Reverse sequence	TGTTAGCAGTGGCTTGATCT	

Reference

Bockmühl DP, Krishnamurthy S, Gerads M, Sonneborn A, Ernst JF (2001) Distinct and redundant roles of the two protein kinase A isoforms Tpk1p and Tpk2p in morphogenesis and growth of *Candida albicans*. Mol Microbiol 42:1243-1257. https://doi.org/10.1046/j.1365-2958.2001.02688.x

Feng QH, Summers E, Guo B, Fink G (1999) Ras signaling is required for serum-induced hyphal differentiation in *Candida albicans*. J Bacteriol 181:6339-6346. https://doi.org/10.1128/JB.181.20.6339-6346.1999

Gillum AM, Tsay EY, Kirsch DR (1984) Isolation of the *Candida albicans* gene for orotidine-5'-phosphate decarboxylase by complementation of *S. cerevisiae ura3* and *E. coli pyrF* mutations. Mol Gen Genet 198:179-182. https://doi.org/10.1007/BF00328721

Mallet L, Renault G, Jacquet M (2000) Functional cloning of the adenylate cyclase gene of *Candida albicans* in *Saccharomyces cerevisiae* within a genomic fragment containing five other genes, including homologues of *CHS6* and *SAP185*. Yeast 16:959-966. https://doi.org/10.1002/1097-0061(200007)16:10<959::AID-YEA592>3.0.CO;2-Q

Zhu C, Liao B, Ye X, Zhou Y, Chen X, Liao M, Cheng L, Zhou X, Ren B (2021) Artemisinin elevates ergosterol levels of *Candida albicans* to synergise with amphotericin B against oral candidiasis. Int J Antimicrob Agents 58:106394. https://doi.org/10.1016/j.ijantimicag.2021.106394
